# Comparative efficacy of two photobiomodulation protocols transcutaneous versus intraoral in the prevention of oral mucositis in head and neck cancer: a randomized study protocol

**DOI:** 10.1007/s10103-026-04977-3

**Published:** 2026-07-29

**Authors:** Waleska Tychanowicz Kolodziejwski, Giovanna Leal Klein Renz, Isabela Busnello de Souza, Maria Luisa Franceschi Coimbra, Luciana Almeida Lopes, Laurindo Moacir Sassi, Melissa Rodrigues de Araujo

**Affiliations:** 1 Federal University of Paraná, Curitiba, Brazil; 2 Nupen Institute, São Carlos, Brazil; 3 Hospital Erasto Gaertner, Curitiba, Brazil

**Keywords:** Low-level light therapy, Oral mucositis, Head and neck neoplasms, Radiotherapy, Photobiomodulation therapy

## Abstract

This study aims to compare the clinical efficacy of transcutaneous photobiomodulation (PBMT) with an 808 nm infrared laser versus intraoral PBMT with a 660 nm red laser in the prevention of oral mucositis (OM). The study is designed to assess whether the transcutaneous approach is non-inferior to the intraoral standard, thereby contributing to the optimization and standardization of PBMT protocols in head and neck cancer (HNC) supportive care. Methods: A randomized pilot study is being conducted with 54 patients (*n* = 27 per group) undergoing radiotherapy (60–70 Gy) for HNC. Participants will be randomly assigned to receive either intraoral PBMT (660 nm, 100 mW, 1 J/point, fluence = 10.20 J/ cm², 10 s, 44 points) or transcutaneous PBMT (808 nm, 100 mW, 1 J/point, fluence = 34.5 J/ cm² (central) and 4.3 J/ cm² (lateral), 10 s, 32 points). Interventions occur three times weekly throughout treatment. The primary outcome is OM severity, graded by WHO scale. Secondary outcomes include OM severity (NCI-CTCAE), pain intensity (Numeric Rating Scale, NRS), ulcerated area extension, registered through digital images, treatment interruptions and unscheduled healthcare utilization. Expected outcomes: the primary hypothesis posits that transcutaneous infrared PBMT is as effective as intraoral red PBMT in mitigating OM severity. Additionally, the transcutaneous approach is expected to offer logistical advantages, including a reduction in application time (80 s vs. 440 s) and improved tolerability in patients with radiation-induced trismus or severe oral pain. Conclusion: This protocol is expected to provide robust evidence to support the most feasible and effective PBMT modality for OM prevention. By optimizing treatment delivery, the study may enhance adherence, preserve nutritional status, and improve the quality of life for patients undergoing oncological therapy.

## Introduction

Head and neck cancer (HNC) encompasses a broad category of various tumor types; approximately 40% of these occur in the oral cavity, 15% in the pharynx, 25% in the larynx, and 20% in other anatomical locations [1, 2]. According to GLOBOCAN data, it is estimated that more than 1.2 million new cases of head and neck cancer will be diagnosed worldwide by 2040 [3].

Antineoplastic treatment (AT) for patients with HNC includes surgical techniques, chemotherapy (CT), radiation therapy (RT), or combination therapy, administered according to the tumor stage and location [4, 5]. For patients with early-stage squamous cell carcinoma, both surgery and radiation therapy yield comparable results in terms of local disease control and overall survival. Following surgery, postoperative radiotherapy, with or without adjuvant chemotherapy, is recommended for patients with pathological risk factors, including perineural invasion, lymphovascular invasion, and positive or narrowly clear surgical margins [6]. RT is an important therapeutic modality for the treatment and control of HNC, as it allows for tumor eradication while preserving the function of normal tissues in the affected region [7], and is used as part of a curative treatment regimen for 75% of patients with head and neck squamous cell carcinoma (HNSCC) [8].

The total radiation dose for curative-intent treatment depends on tumor location and type, typically ranging from 50 to 70 Gy in conventional radiotherapy regimens. In most cases, this dose is delivered in fractions of 1.8 to 2.0 Gy per day, five days a week, over a period of five to seven weeks [9].

Side effects may occur with these treatments; in the oral cavity, the acute effects of RT include oral mucositis, changes in saliva viscosity and volume, dysgeusia, candidiasis, and limited mobility [5]. Chronic effects include neuropathy, atrophy of the facial muscles and salivary glands, halitosis, dysphagia, dysphonia, osteoradionecrosis, xerostomia, hyposalivation, dental caries, and periodontal disease [5].

Oral mucositis (OM) is the most common and challenging complication of radiation therapy or chemoradiotherapy (CRT) in patients undergoing cancer treatment, most commonly found in nearly 100% of patients undergoing high-dose chemotherapy and hematopoietic stem cell transplantation and in 75% to 90% of patients treated for head and neck malignancies [10, 11, 12, 13, 14]. Complications such as painful mouth ulcers and difficulty eating or swallowing are characteristic of OM. The pain and severe dysphagia resulting from OM can lead to significant weight loss in the patient, with a general worsening of their overall condition, and often cause temporary interruption of radiation therapy, which has been shown to reduce its efficacy [14, 15].

In this context, photobiomodulation therapy (PBMT), also known as low-level laser therapy, has emerged as an important adjunctive tool in the prevention and management of OM [16, 17]. PBMT uses light in the red and near-infrared wavelength ranges to modulate cellular processes without causing thermal damage to tissues. The light is absorbed primarily by mitochondrial cytochrome c oxidase and light-sensitive ion channels, triggering increased ATP production, controlled release of reactive oxygen species and nitric oxide, as well as modulation of intracellular calcium [18, 19, 20]. These events activate transcription factors related to cell survival, proliferation, migration, and the synthesis of new proteins, resulting in anti-inflammatory, analgesic, and tissue repair effects [18, 19, 20, 21, 22]. In contexts of oxidative stress, such as during RT, PBMT tends to reduce oxidative stress and inflammation, while simultaneously stimulating mucosal regeneration [19, 21, 22].

In the management of mucositis induced by anticancer therapy, PBMT is used for both preventive and curative purposes, following protocols recommended by international guidelines such as Multinational Association of Supportive Care in Cancer/International Society of Oral Oncology (MASCC/ISOO), particularly in patients undergoing RT and CRT of the head and neck [16, 17, 21]. Preventively, intraoral or transcutaneous application is initiated at the start of treatment and performed regularly (usually daily or several times a week), reducing the incidence, severity, and duration of mucositis, as well as pain intensity and the need for opioids [16, 17, 21, 22, 23]. When lesions are already established, PBMT accelerates healing, shortens the duration of severe mucositis, and improves swallowing, nutrition, and quality of life [16, 22, 24].

Therefore, although there are already clinical trials comparing intraoral and transcutaneous photobiomodulation protocols for the prevention and management of OM in different oncological settings, these studies use heterogeneous parameters and application frequencies, which still hinder the establishment of a standardized protocol for head and neck cancer. Thus, a critical unmet need remains in determining the optimal PBMT delivery strategy—namely, whether intraoral red laser or transcutaneous infrared laser is more appropriate for the prevention and mitigation of radiotherapy-induced OM. Notably, direct comparative evidence between these approaches remains lacking. This randomized study aims to compare the efficacy of these two modalities in patients with head and neck cancer, providing evidence to support the standardization of PBMT protocols and to optimize supportive care during anticancer treatment.

The primary objective of this study is to compare the clinical efficacy of two PBMT protocols: TC PBMT using an 808 nm infrared laser and IO PBMT using a 660 nm red laser for the prevention of OM.

## Materials and methods

### Study Design and Setting 

This pilot study, conducted at a reference oncology center in Paraná, Brazil, compares two distinct PBMT protocols used clinically to prevent OM in patients undergoing radiotherapy for head and neck cancer: TC PBMT (infrared laser) versus IO PBMT (red laser). The two protocols differ in wavelength, fluence, energy per session, number of irradiation points, and application geometry, reflecting the intrinsic characteristics of each available clinical modality. The study aims to compare the overall clinical performance of each protocol. The protocol follows Standard Protocol Items for Recommendations for Interventional Trials (SPIRIT) guidelines. Participants are randomly allocated into one of two intervention: the TC group or the IO group.

### Ethical considerations and registration

The study was approved in October 2024 by the Human Research Ethics Committee of Hospital Erasto Gaertner, under registration number 7.072.369. This study was registered in the Brazilian Registry of Clinical Trials (ensaiosclinicos.gov.br) in June 2025, under registration number RBR-9brv7dd. The participants are informed that they can withdraw from the study at any time for any reason, if they desire.

## Sample size

The sample size was determined based on a non-inferiority design, assuming that transcutaneous infrared PBMT would demonstrate clinical efficacy comparable to intraoral red PBMT in the prevention of OM. The non-inferiority margin was defined as Δ = 0.5 points on the WHO scale, based on the differences observed between CT and IO protocols in direct comparative studies [25, 26]. Calculations were performed using the ClinCalc platform to compare two independent means, assuming a significance level (α) of 0.025 (one-tailed test) and a statistical power (1 - β) of 80%. To account for a predicted 10% attrition rate (dropouts), the final sample size was established at 54 participants (*n* = 27 per group). Participants will be recruited through consecutive sampling to ensure a representative flow of patients at the study site.

## Randomization and blinding

Participants will be randomized (1:1) to TC or IO groups via Random.org (*n* = 54). The research team will perform sequential, simple randomization without blocks or stratification. Due to the distinct application methods and wavelengths (red vs. infrared), double-blinding is unfeasible. Moreover, outcome assessors responsible for WHO and NCI-CTCAE oral mucositis grading cannot be blinded, as the clinical team involved in treatment delivery will also perform the evaluations. Therefore, this is an open-label trial, and the lack of blinding represents a potential limitation of the study. To minimize potential detection bias, examiners will undergo calibration before study initiation, standardized assessment criteria will be applied, and photographic documentation will support mucositis evaluation.

## Inclusion and exclusion criteria

Eligibility depends on clinical and staging requirements, summarized in Box 1.

**Box 1.** Inclusion and exclusion criteria.

**Table:** 

Inclusion Criteria	Exclusion Criteria
Patients diagnosed with malignant tumors in the head and neck region	Previous radiation therapy to the head and neck region
Who will start RT or CRT	Severe or uncontrolled periodontal disease
Both genders	Patients undergoing topical oral anti-inflammatory medication therapy (e.g., Omcilon-A Orabase^®^ [triamcinolone acetonide])
Ages 18 and older	
Informed Consent Form (ICF) signed	

## Clinical and treatment-related variables

Prophylactic PBMT will be administered thrice weekly during radiation therapy. Following institutional standards, all patients will undergo intensity-modulated radiation therapy (IMRT). The prescribed radiation regimen consists of total doses ranging from 60 to 70 Gy, delivered in 30 to 35 fractions of 2 Gy (200 cGy) per fraction, five days a week.

Following a pragmatic design, patients on concurrent chemotherapy will be included. As chemotherapy significantly impacts OM risk, it will be recorded and treated as a covariate in multivariable regression analyses to adjust for potential confounding on outcomes.

The standardized PBMT schedule was set for Monday, Wednesday, and Friday during all weeks of RT, ensuring a maximum interval of two days between sessions and coverage of at least three consecutive RT fractions. Three times per week (rather than daily) was based on the operational feasibility of the referral oncology service (patient flow and researcher availability), evidence from randomized clinical trials demonstrating that the efficacy of three-times-weekly PBMT protocols is comparable to daily frequency in SCC populations [22], and protocols approved by the MASCC/ISOO guidelines that include alternate frequencies [17].

### Protocols

#### Transcutaneous PBMT

**Fig. 1:**
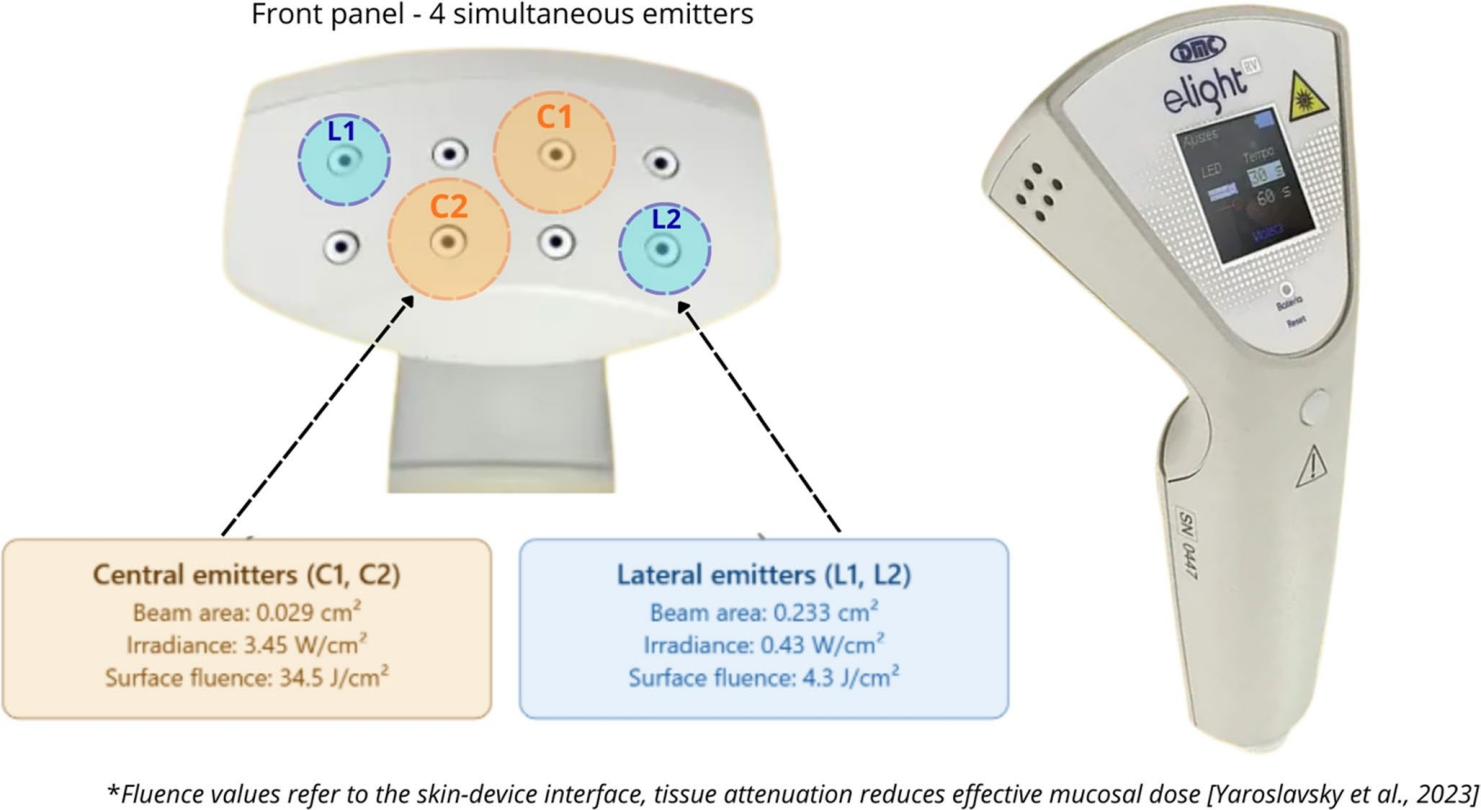
Cluster device showing the four simultaneous emitters and dosimetric parameters of central (C1, C2) and lateral (L1, L2) emitters

TC PBMT utilizes the E-light IRL cluster (DMC^®^, São Carlos, SP, Brazil; hardware v. 2.0 101). Although the device incorporates dual-wavelength emitters (660 nm and 808 nm), only the 808 nm infrared (IR) emitters will be utilized for this study. These emitters consist of Aluminum Gallium Arsenide (AlGaAs) semiconductor diodes. The cluster comprises four IR emitters operating simultaneously, each with an output power of 100 mW. Each emitter will deliver 1 J/spot in 10 s. Beam characteristics are described as follows (Fig. 1):

- Central Emitters (*n* = 2): beam area of 0.029 cm², resulting in an irradiance of 3.45 W/cm² and a fluence of 34.5 J/cm² (1 J per emitter).
- Lateral Emitters (*n* = 2): beam area of 0.233 cm², resulting in an irradiance of 0.43 W/cm² and a fluence of 4.3 J/cm² (1 J per emitter).

Detailed dosimetric parameters are summarized in Table 1.

The fluence values of central (34.5 J/cm²) and lateral emitters (4.3 J/cm²) refer to the skin-device interface; effective fluence at the oral mucosa will be considerably lower due to tissue attenuation, with a reported 10 to 50 fold reduction depending on total tissue thickness [27], which is acknowledged as a limitation of the TC protocol.

Application Technique: Eight anatomical points will be irradiated in contact mode. Given the simultaneous firing of the four emitters, each application point receives a total of 4 J (1 J/ emitter), totaling 32 J/ session (80 s cumulative).

**Fig. 2:**
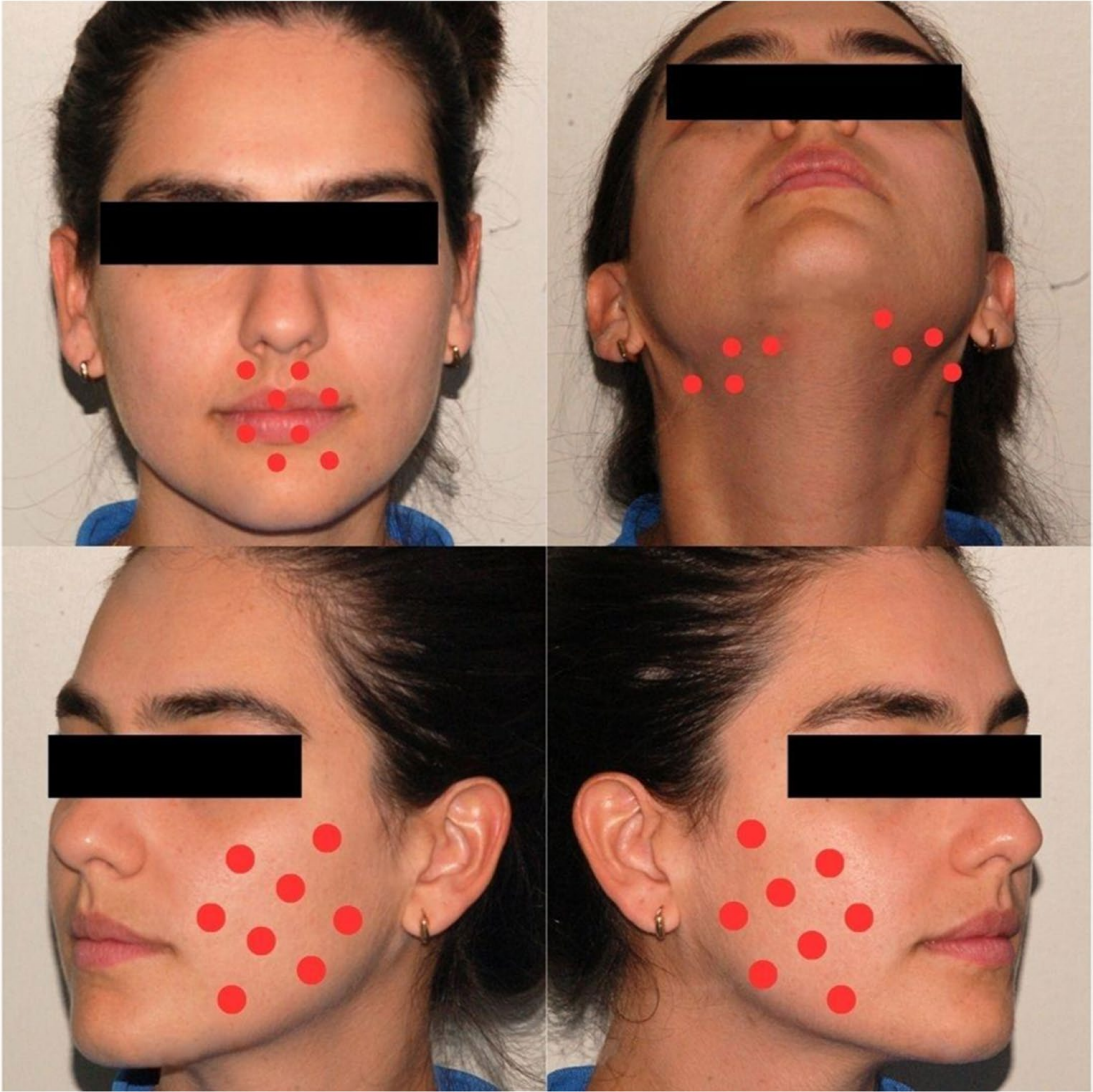
Transcutaneous application points: the orbicularis oris muscle, the floor of the mouth and left and right buccinator muscle

The anatomical mapping for TC PBMT includes (Fig. 2):

1. Buccinator muscles: eight points/side, right and left.
2. External orbicularis oris: eight points (four superior and four inferior).
3. Submandibular glands: four points (two/side, right and left).

The selection of 808 nm is supported by its positioning within the near-infrared optical therapeutic window (700–1100 nm), which minimizes absorption by hemoglobin and water, thereby maximizing tissue penetration depth [28]. Site-specific fluence estimates based on Monte Carlo simulations for 850 nm light spectrally close to the present device demonstrate considerable variation by anatomical site: 0.4–10.0 mW/cm² at the cheek, 2.4–35.5 mW/cm² at the mandible angle, and 5.6–36.3 mW/cm² at the lip, depending on total tissue thickness [27]. For the lateral tongue, irradiation is performed via submandibular application points, with light traversing skin, subcutaneous tissue, and muscular layers before reaching the target mucosa. The soft palate was not included among TC application points due to its anatomically unfavorable position for transcutaneous light delivery, which is acknowledged as a coverage limitation of the TC protocol.

### Intraoral PBMT

IO PBMT will be administered using the Therapy EC device (DMC^®^, São Carlos, SP, Brazil). This system utilizes an Aluminum Gallium Indium Phosphide (AlGaInP) semiconductor diode laser featuring a single-emitter laser (0.098 cm²). The procedure will be performed in contact mode using a visible red wavelength of 660 nm.

The device operates at an output power of 100 mW, resulting in an irradiance of 1.02 W/cm². Each application point will receive 1 J of energy over a 10-second irradiation interval, corresponding to a fluence of 10.20 J/cm² (Table 1).

Application Technique: A 44-point protocol covers the buccal mucosa [29]. The anatomical distribution is defined as follows:

- Buccal mucosa: 16 points (8/ side).
- Labial mucosa: 8 points (4 superior and 4 inferior).
- Buccal folds: 6 points (3/ side).
- Tongue: 9 points (4 on each lateral border and 1 on the tip).
- Soft palate: 3 points.
- Labial commissures: 2 points (1/ side).

**Fig. 3:**
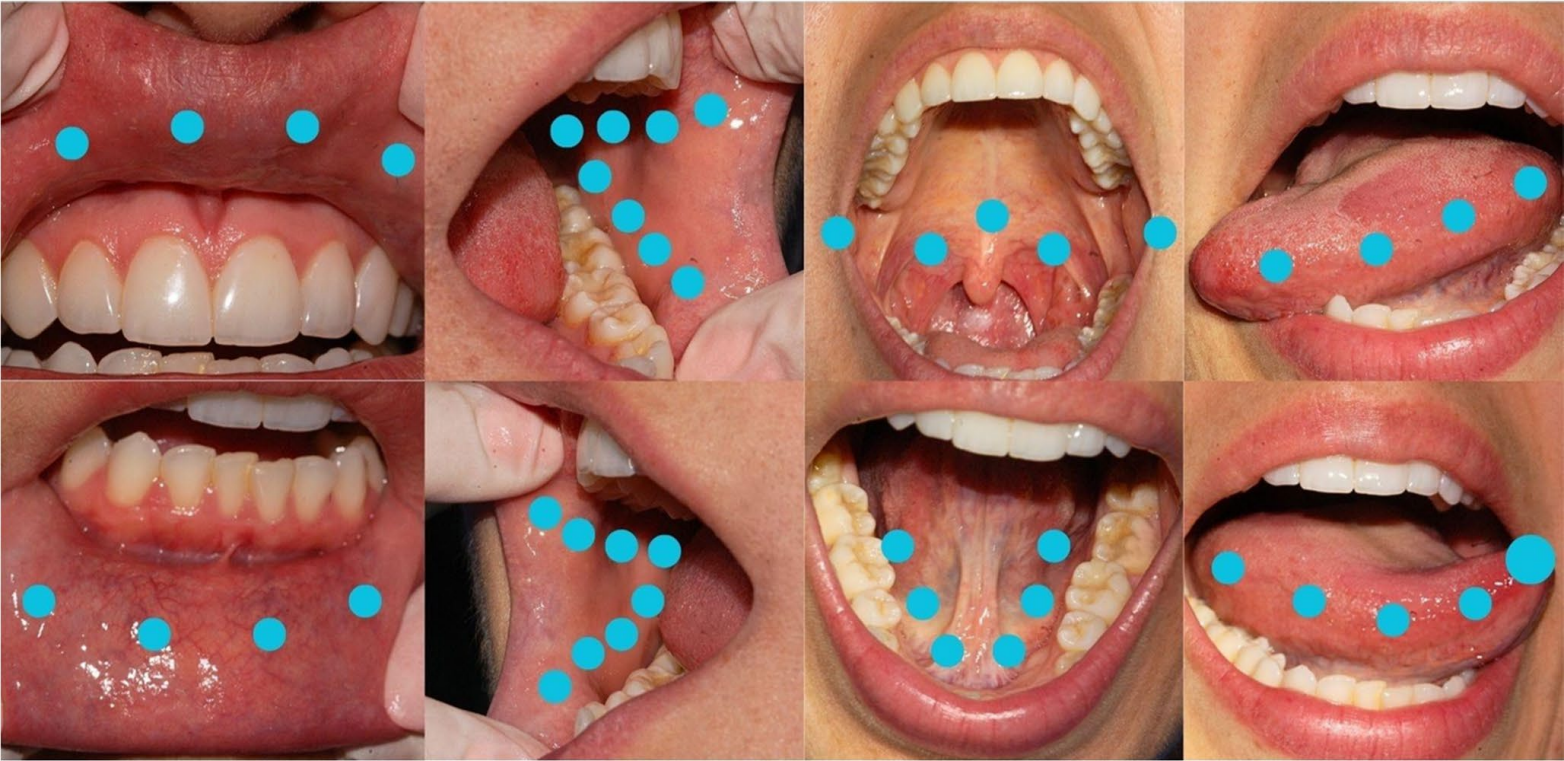
Intraoral PBMT points application sites located in non-keratinized mucosal: superior and inferior oral, and left and right buccal mucosa, soft palate, floor of the mouth, left and right tongue borders

Total energy is 44 J/ session, lasting 440 s (7 min and 20 s). The clinical mapping of these points is illustrated in Fig. 3.

Three calibrated examiners will perform all clinical assessments; however, blinding will not be feasible in this study due to the visually distinguishable nature of the two PBMT protocols.

For both TC and IO protocols, PBMT will not be applied directly to the gross tumor volume (GTV), as defined in the IMRT planning records available in the patient’s medical record. In cases where any predetermined application point coincides with the GTV, the point will be repositioned or omitted, and this decision will be recorded on the data collection form.

**Table 1:** Parameters of intraoral and transcutaneous photobiomodulation

PARAMETER	INTRAORAL	TRANSCUTANEOUS
Wavelength (nm)	660	808
Laser type	AlGaInP diode laser	AlGaAs diode laser (4-emitter cluster)
Power per emitter (mW)	100	100
Total cluster power (mW)	–	400
Spot size (cm²)	0.098	Central emitters: 0.029 Lateral emitters: 0.233
Irradiance (W/cm²)	1.02	Central: 3.45 Lateral: 0.43
Energy per point (J)	1	1
Fluence (J/cm²)	10.20	Central: 34.5 Lateral: 4.3
Time per point (s)	10	10
Number of points per session	44	8 applications (4 emitters, 32 points)
Total energy per session (J)	44	32
Total irradiation time per session (s)	440	80
Application mode	Contact	Contact

### Standard supportive care protocol

All participants will receive standardized oral care per MASCC/ISOO guidelines [17]. This supportive regimen, implemented uniformly across both study arms, includes professional oral hygiene protocols, weekly dental assessments, supervised use of non-alcoholic mouthwashes, and systematic lip hydration.

Upon clinical diagnosis of ulcerative OM, localized therapeutic PBMT (660 nm, 2 J/spot, 100 mW, 20s, 20.41 J/cm²) will be initiated until complete repair [30]. When a preventive application point coincides with an ulcerated region, therapeutic irradiation will replace the preventive dose at that point. Total therapeutic sessions and cumulative energy will be recorded as potential confounders.

Procedures follow safety standards and manufacturer recommendations, including wavelength-specific protective eyewear for patients and operators. To prevent cross-contamination, handpieces will be disinfected with 70% isopropyl alcohol and covered with disposable PVC film.

Although PBMT is low-risk, participants will be monitored for adverse effects, (e.g., transient discomfort, thermal sensation, or erythema). Any event will be documented, and therapy will be discontinued if reported or observed.

### Data collection

All study data, including research-specific instruments and clinical forms, will be stored in both physical file folders and on a password-protected personal computer under the direct responsibility of the principal investigators. To ensure confidentiality, participants will be de-identified using unique numerical codes.

At baseline, a Case Report Form (CRF) will record demographics (age, sex), oncological diagnosis, and comorbidities. During each PBMT session, participants will undergo systematic clinical evaluation. Any clinical changes, adverse reactions, or OM progression will be documented on a standardized longitudinal follow-up form to monitor the incidence and trajectory of oral lesions throughout treatment.

The primary clinical endpoint:

1. OM severity

Will be assessed by a trained examiner at each clinical visit. To ensure a comprehensive evaluation of both functional and clinical aspects, severity will be graded according to the established criteria of the World Health Organization (WHO) and the National Cancer Institute Common Terminology Criteria for Adverse Events (NCI-CTCAE) [31] (Table 2).

The WHO scale will be used to integrate objective clinical signs (erythema and ulceration) with the patient’s functional ability to maintain oral intake. Simultaneously, the NCI-CTCAE scale will provide a standardized grading of symptomatic and clinical toxicity. The detailed scoring criteria for both instruments are summarized in Table 2.

**Table 2:** Classification of OM according to the WHO and NCI scales

Scales	WHO	NCI
0	Normal mucosa	Normal mucosa
1	Erythema	Erythema
2	Ulcer, normal diet	Non-contiguous ulcers up to 1.5 cm in diameter
3	Ulcer, liquid diet	Contiguous ulcers larger than 1.5 cm in diameter
4	Ulcer, unable to tolerate oral intake	Ulcers with necrosis and bleeding

2.) Topographic mapping of oral lesions

To accurately track the progression and resolution of OM, a systematic topographic mapping of the oral cavity will be performed during each clinical evaluation. The presence, extension, and anatomical location of erythema and ulcerative lesions will be documented using a standardized oral cavity map.

The assessment will encompass the following anatomical sites:

- Non-keratinized mucosa: Buccal mucosa, labial mucosa, ventral/ lateral tongue, soft palate, and floor of the mouth.
- Keratinized mucosa: Dorsum of tongue and hard palate.

The examiner will record specific sub-sites and lesion surface area, enabling comparative analysis of TC versus IO PBMT efficacy in reaching anatomically shielded regions, such as the oropharynx and tongue base. The ulcerated area will be quantified through topographic mapping, estimation of the lesion’s extent in cm² using a clinical ruler, and standardized digital intraoral photography.

3.)  Pain

The Numerical Rating Scale (NRS) for pain consists of an 11-point scale ranging from 0 to 10, where 0 represents “no pain/difficulty” and 10 indicates the “worst pain/difficulty imaginable.” Patients are asked to choose a whole number that best describes the intensity of the pain or difficulty they are experiencing, facilitating a quick and objective quantification of the painful experience [32].

Assessments will be conducted at baseline, immediately following each PBMT session, and during all subsequent follow-up visits. This longitudinal monitoring aims to evaluate the patient’s symptomatic response to the intervention over time. Participants will be instructed to mark the point on the scale that most accurately represents their current status for each evaluated parameter, providing a quantitative measure of subjective treatment impact.

### Primary outcome

The primary outcome is OM severity, graded via the WHO scale [ 31], comparing TC versus IO PBMT groups in patients undergoing RT or CRT. Comparative analyses will be performed between transcutaneous and intraoral PBMT groups.

### Secondary outcomes

Compare IO and TC PBMT regarding:

- OM severity (NCI-CTCAE);
- Pain intensity (NRS);
- Ulcerated area extension (grades 2–4) will be registered with digital images;
- Treatment interruptions (unplanned RT/CRT modifications);
- Unscheduled healthcare utilization (emergency visits or hospitalizations due to OM complications).

This study explores PBMT delivery routes to standardize application parameters and optimize supportive care for patients with head and neck malignancies.

### Statistical analysis

Analyses will be performed using Jamovi software (version 2.6.44). Normality will be assessed using the Shapiro-Wilk test, and variables will be described using frequencies and percentages (categorical variables) or means, standard deviations, and medians with interquartile ranges (continuous variables). Baseline comparability between groups will be assessed using Student’s t-test or the Mann-Whitney U test (continuous variables) and the chi-square test or Fisher’s exact test (categorical variables).

The primary outcome, oral mucositis severity (WHO scale), will be analyzed longitudinally using a mixed-effects ordinal logistic regression model to account for the repeated measurements. The model will include treatment group, assessment time, and the treatment vs. time interaction as fixed effects, with participant included as a random effect to account for within-subject correlation. Total radiotherapy dose, tumor location, and concomitant chemotherapy will be included as covariates.

Pain intensity (NRS) will be analyzed longitudinally using an appropriate mixed-effects regression model according to its distribution. The primary analysis will follow the intention-to-treat principle. Missing outcome data will not be imputed; instead, the mixed-effects ordinal logistic regression model will incorporate all available repeated measurements and account for incomplete observations over time under the assumption that data are missing at random.

The primary objective is to determine whether transcutaneous infrared PBMT is non-inferior to intraoral red PBMT regarding oral mucositis severity. Non-inferiority will be assessed using the estimated treatment effect obtained from the longitudinal mixed-effects model and interpreted according to the prespecified non-inferiority margin of 0.5 WHO grade.

### Treatment adherence and discontinuation

Patient adherence to RT and PBMT will be monitored. While interruptions are not initial exclusion criteria, discontinuation is defined as:

- RT discontinuation: Cessation before the full dose (medical decision or refusal). Such participants will be excluded from follow-up;
- PBMT discontinuation: missing two or more consecutive sessions or complete intervention cessation, including missed appointments or loss to follow-up, will not result in exclusion from the study. These participants will be treated in the analysis according to the intention-to-treat (ITT) principle, with all data collected up to the time of withdrawal included in the analyses. Withdrawal from PBMT will be recorded as an adherence variable, allowing for an exploratory analysis of the relationship between the number of sessions completed and clinical outcomes. A per-protocol analysis, including only participants who completed at least 80% of the scheduled PBMT sessions, will be performed as a sensitivity analysis to verify the robustness of the results obtained in the ITT analysis.

### Participant timeline

**Fig. 4:**
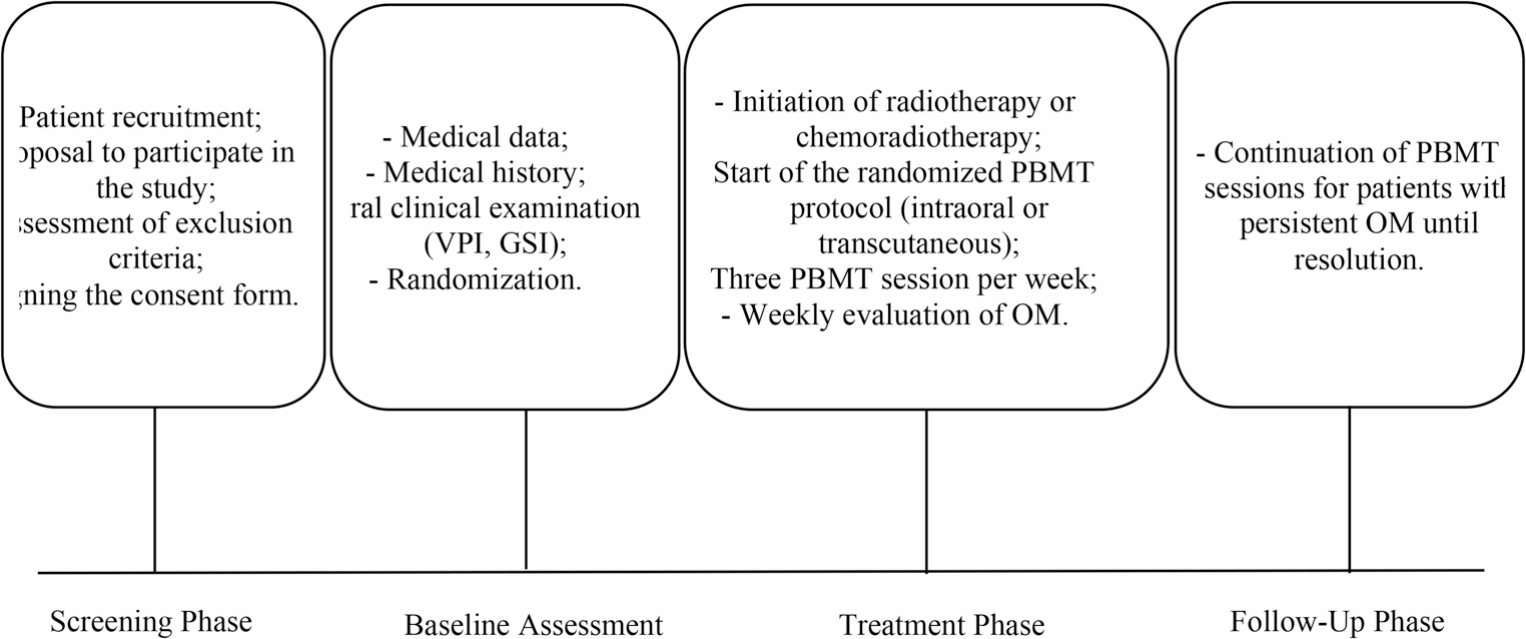
Research stages. *VPI: visible plaque index; GSI: gingival bleeding index

The participant timeline outlines the research stages, from recruitment and clinical assessment to the treatment protocol and follow-up phase (Fig. 4).

### Study timeline

The expected duration of this clinical trial is 19 months. This timeline allows sufficient time for patient recruitment, data collection, and analysis.

### Expected results

This study generates comparative data on TC (infrared) versus IO (red) PBMT efficacy for OM prevention during head and neck radiotherapy.

The primary hypothesis is that TC PBMT is non-inferior or superior to IO PBMT in preventing OM. Furthermore, TC PBMT improves compliance in patients with trismus or limited mouth opening, as it obviates the need for prolonged intraoral access during treatment.

Regarding the outcomes of pain and ulcerated area extent, transcutaneous PBMT is expected to demonstrate efficacy equivalent to or superior to that of intraoral PBMT, providing significant relief of pain symptoms through its greater tissue penetration depth and modulation of inflammation.

Furthermore, the results support the development of standardized, evidence-based protocols for the implementation of PBMT in clinical settings, ensuring the consistent and reliable application of this therapy across various patient subgroups. Consequently, this research will provide baseline data for future proposals, serving as a foundation for larger-scale multicenter trials and longitudinal evaluations of the efficacy and safety of photobiomodulation.

## Discussion

Mucositis remains a common and debilitating complication of CRT in patients with head and neck cancer, associated with severe pain, worsening nutritional status, treatment interruptions, and a significant decline in quality of life, even with modern RT protocols and supportive care [22, 23, 33].

Recent systematic reviews and meta-analyses have established a high level of evidence confirming that PBMT effectively reduces the incidence, duration, and severity of OM, as well as associated pain, in the HNC population. These therapeutic effects typically manifest between the second and third weeks of RT and persist throughout the treatment course [23, 34]. Reflecting this robust evidence, the MASCC/ISOO clinical practice guidelines updated their recommendations to strongly favor the use of intraoral PBMT for the prevention of OM in patients undergoing either RT alone or concurrent CRT [17, 23, 35]. While the intraoral route is currently the clinical standard, the exploration of alternative delivery modalities such as transcutaneous application remains a critical frontier in optimizing patient comfort and clinical efficiency.

Evidence from clinical trials highlights that both daily and thrice-weekly prophylactic PBMT regimens significantly attenuate severe oropharyngeal pain and reduce the requirement for opioid and non-opioid analgesics [22, 33, 36, 37]. Beyond pain management, recent data by Hu et al. [38] have demonstrated that daily PBMT not only mitigates oropharyngeal discomfort but also results in a lower incidence of enteral feeding tube insertions. These findings suggest that PBMT plays a pivotal role in preserving nutritional autonomy and functional status, ultimately leading to superior health-related quality of life (HRQoL) during aggressive oncological treatment. By maintaining the integrity of the oral and oropharyngeal mucosa, PBMT facilitates the maintenance of oral intake, preventing the common trajectory of weight loss and dehydration associated with chemoradiotherapy [22, 33, 34, 36, 37, 38].

While low-power AlGaInP semiconductors have yielded particularly favorable clinical outcomes, as highlighted by Shen et al.[23] in their systematic review and meta-analysis, there is considerable heterogeneity in wavelength, fluence, frequency, and mode of application, which still makes it difficult to establish a “gold standard” protocol [22]. Studies comparing daily versus alternate-day frequency reinforce that more intensive protocols tend to provide better control of OM and pain [22].

Recent data reinforce the interest in transcutaneous PBMT, due to its greater convenience and reduced discomfort. A randomized trial of prophylactic transcutaneous PBMT in HNC patients demonstrated a delay in the onset of OM, less pain, reduced analgesic use, and improved quality of life, with no negative impact on 1-year survival [39]. Trials in chemotherapy and hematopoietic transplantation suggest that approaches combining intraoral and transcutaneous methods may enhance prevention while maintaining good tolerability [36, 39]. Nevertheless, head-to-head studies in HNC with rigorously standardized parameters are still lacking.

By directly comparing intraoral PBMT (red) and transcutaneous PBMT (infrared) using clearly defined parameters and for prophylactic application during RT, the study aligns with current priorities: reducing protocol variability, clarifying whether the transcutaneous route may be non-inferior to the intraoral route in terms of efficacy, based on the ability of infrared wavelengths to penetrate soft tissues and reach deep structures, although the intensity of the photons undergoes exponential attenuation, evidence suggests that the energy deposited transcutaneously is capable of reaching the oral mucosa and tongue tissues, although the bone density of the palate may represent a greater barrier to penetration, while offering greater comfort and logistical feasibility, and exploring relevant clinical outcomes, unplanned RT interruptions, the need for nutritional support, and quality of life [ 40, 41, 42].

Recent literature comparing IO and TC PBMT confirms that while both modalities are clinically effective for the prevention and management of OM, they exhibit distinct optical delivery profiles and impact the patient experience differently. A critical consideration in PBMT is that light intensity attenuates exponentially as it traverses biological tissue. Consequently, the intraoral route offers a direct advantage by delivering photons directly to the target irradiated mucosa. In contrast, the efficacy of the transcutaneous route is highly dependent on the optimization of laser parameters, specifically wavelength (favoring the “optical window” of near-infrared), irradiance, and exposure time to ensure that a sufficient therapeutic dose reaches the deeper mucosal layers [25, 42, 43]. Trials in hematopoietic stem cell transplantation and animal models reinforce that intraoral PBMT tends to promote a more localized effect and faster healing, although transcutaneous PBMT also contributes to epithelial recovery when used with appropriate parameters [42, 43].

On the other hand, Multicenter clinical trials in head and neck cancer and pediatric populations indicate that transcutaneous protocols may be as effective as intraoral ones in reducing the incidence, severity, and duration of OM, with no significant differences in the time to onset of ulcerated lesions or in overall mucositis scores [25, 42, 43, 44, 26]. In a randomized study of oral cavity and oropharyngeal carcinoma, transcutaneous PBMT using an 810/980 nm superpulsed laser demonstrated efficacy equivalent to intraoral PBMT across all grades of mucositis, with some additional functional benefits, such as reduced difficulty swallowing and less loss of taste at critical points in treatment [25, 26]. Similar results were reported in pediatric oncology patients, in whom transcutaneous PBMT was considered comparable to intraoral PBMT in terms of OM severity, duration of lesions, and functional impact, with a slight difference in pain at only one follow-up point [44].

Factors such as patient comfort, logistics, and treatment time also distinguish these modalities. Intraoral PBMT, although it focuses energy directly on the most affected sites (tongue margins, buccal mucosa, soft palate), is more dependent on radiation-induced trismus, which may cause discomfort in patients with severe pain and requires longer session times and closer professional-patient proximity [17, 41, 42, 43]. In contrast, transcutaneous protocols tend to be faster, technically simpler, well-tolerated, and require less application time, which translates to greater feasibility for high-volume services and potential for home use [17, 37, 42, 43]. In hematopoietic transplantation, for example, the duration of the transcutaneous session was approximately one-quarter of the intraoral session, while maintaining similar efficacy in preventing OM [43].

Given this scenario, hybrid or staged strategies are being developed, combining intraoral and transcutaneous PBMT or selecting the approach based on anatomical risk and clinical conditions. Studies using combined protocols in head and neck cancer and in patients undergoing chemotherapy alone show that the combination of intraoral and transcutaneous approaches can further reduce OM scores, improve salivary flow and quality of life, without increasing adverse events [34, 45]. Recent guidelines and reviews suggest that the choice between intraoral PBMT and transcutaneous PBMT should consider not only efficacy but also patient adherence, the pattern of radiation therapy fields, and the need to cover deep or hard-to-reach areas, such as the oropharynx and base of the tongue [16, 17].

## Conclusion

The current literature confirms PBMT as an effective and safe intervention for preventing and mitigating OM in patients with head and neck cancer undergoing RT or CRT; however, there is still a lack of robust evidence comparing administration routes. In this context, the present study directly contributes to the standardization of parameters and to the selection of the most effective and feasible modality in clinical practice, with potential impact on the continuity of cancer treatment, pain control, and quality of life. Ultimately, these findings will support the integration of more efficient supportive care protocols.

## Data Availability

No datasets were generated or analysed during the current study.
